# A Logical Model Provides Insights into T Cell Receptor Signaling

**DOI:** 10.1371/journal.pcbi.0030163

**Published:** 2007-08-24

**Authors:** Julio Saez-Rodriguez, Luca Simeoni, Jonathan A Lindquist, Rebecca Hemenway, Ursula Bommhardt, Boerge Arndt, Utz-Uwe Haus, Robert Weismantel, Ernst D Gilles, Steffen Klamt, Burkhart Schraven

**Affiliations:** 1 Max Planck Institute for Dynamics of Complex Technical Systems, Magdeburg, Germany; 2 Institute of Immunology, Otto-von-Guericke University, Magdeburg, Germany; 3 Institute for Mathematical Optimization, Otto-von-Guericke University, Magdeburg, Germany; Utrecht University, The Netherlands

## Abstract

Cellular decisions are determined by complex molecular interaction networks. Large-scale signaling networks are currently being reconstructed, but the kinetic parameters and quantitative data that would allow for dynamic modeling are still scarce. Therefore, computational studies based upon the structure of these networks are of great interest. Here, a methodology relying on a logical formalism is applied to the functional analysis of the complex signaling network governing the activation of T cells via the T cell receptor, the CD4/CD8 co-receptors, and the accessory signaling receptor CD28. Our large-scale Boolean model, which comprises 94 nodes and 123 interactions and is based upon well-established qualitative knowledge from primary T cells, reveals important structural features (e.g., feedback loops and network-wide dependencies) and recapitulates the global behavior of this network for an array of published data on T cell activation in wild-type and knock-out conditions. More importantly, the model predicted unexpected signaling events after antibody-mediated perturbation of CD28 and after genetic knockout of the kinase Fyn that were subsequently experimentally validated. Finally, we show that the logical model reveals key elements and potential failure modes in network functioning and provides candidates for missing links. In summary, our large-scale logical model for T cell activation proved to be a promising in silico tool, and it inspires immunologists to ask new questions. We think that it holds valuable potential in foreseeing the effects of drugs and network modifications.

## Author Summary

T-lymphocytes are central regulators of the adaptive immune response, and their inappropriate activation can cause autoimmune diseases or cancer. The understanding of the signaling mechanisms underlying T cell activation is a prerequisite to develop new strategies for pharmacological intervention and disease treatments. However, much of the existing literature on T cell signaling is related to T cell development or to activation processes in transformed T cell lines (e.g., Jurkat), whereas information on non-transformed primary T cells is limited. Here, immunologists and theoreticians have compiled data from the existing literature that stem from analysis of primary T cells. They used this information to establish a qualitative Boolean network that describes T cell activation mechanisms after engagement of the TCR, the CD4/CD8 co-receptors, and CD28. The network comprises 94 nodes and can be extended to facilitate interpretation of new data that emerge from experimental analysis of T cell activation. Newly developed tools and methods allow in silico analysis, and manipulation of the network and can uncover hidden/unforeseen signaling pathways. Indeed, by assessing signaling events controlled by CD28 and the protein tyrosine kinase Fyn, we show that computational analysis of even a qualitative network can provide new and non-obvious signaling pathways which can be validated experimentally.

## Introduction

Understanding how cellular networks function in a holistic perspective is the main purpose of systems biology [1]. Dynamic models provide an optimal basis for a detailed study of cellular networks and have been applied successfully to cellular networks of moderate size [2–5]. However, for their construction and analysis they require an enormous amount of mechanistic details and quantitative data which, until now, has been often lacking in large-scale networks. Therefore, there has been considerable effort to develop methods based exclusively on the often well-known network topology [6,7]. One may distinguish between studies on the statistical properties of graphs [8–10] and approaches aiming at predicting functional or dysfunctional states and modes. For the latter, a large corpus of methods has been developed for metabolic networks mainly relying on the constraints-based approach [11,12]. However, for signaling networks, methods facilitating a similar functional analysis—including predictions on the outcome of interventions— have been applied to a much lesser extent [6].

Here we demonstrate that capturing the structure of signaling networks by a recently introduced logical approach [13] allows the analysis of important functional aspects, often leading to predictions that can be verified in knock-out/perturbation experiments. Logical networks have until now been used for studying artificial (random) networks [14] or relatively small gene regulatory networks [15–18]. In contrast, herein we study a large-scale signaling network, structured in input (e.g., receptors), intermediate, and output (e.g., transcription factors) layers. Compared with gene regulatory networks, the behavior of signaling networks is mainly governed by their input layer, shifting the interest to input–output relationships. Addressing these issues requires partially different techniques, as compared with gene regulatory networks. We use a special and intuitive representation of logical networks (called *logical interaction hypergraph* (LIH); see Methods), which is well-suited for this kind of input–output analysis. By applying *logical steady state analysis,* one may predict how a combination of signals arriving at the input layer leads to a certain response in the intermediate and the output layers. Additionally, this approach facilitates predictions of the effect of interventions and, moreover, allows one to search for interventions that repress or provoke a certain logical response [13]. Furthermore, each logical network has a unique underlying interaction graph from which other important network properties such as feedback loops, signaling paths, and network-wide interdependencies can be evaluated.

Importantly, we consider here a logical model to be constructed by collecting and integrating well-known *local* interactions (e.g., a kinase phosphorylates an adaptor molecule). The logical model is then employed to derive *global* information (e.g., stimulation of a receptor leads to the activation of a certain transcription factor via several logical connections). Thus, the available data on the global network behavior is not used to construct the model; instead, it is used to *verify* the model. The model may then be employed to *predict* global responses that have not yet been studied experimentally.

Here, we apply the logical framework to a carefully constructed model of T cell receptor (TCR) signaling. T-lymphocytes play a key role within the immune system: cytotoxic, CD8^+^, T cells destroy cells infected by viruses or malignant cells, and CD4^+^ T helper cells coordinate the functions of other cells of the immune system [19]. The importance of T cells for immune homeostasis is due to their ability to specifically recognize foreign, potentially dangerous, agents and, subsequently, to initiate a specific immune response. T cell reactivity must be exquisitely regulated as either a decrease (which weakens the defense against pathogens with the consequence of immunodeficiency) or an increase (which can lead to autoimmune disorders and leukemia) can have severe consequences for the organism.

T cells detect foreign antigens by means of the TCR, which recognizes peptides only when presented upon MHC (Major Histocompatibility Complex) molecules. The peptides that are recognized by the TCR are typically derived from foreign (e.g., bacterial, viral) proteins and are generated by proteolytic cleavage within so-called antigen presenting cells (APCs). Binding of the TCR to peptide/MHC complexes and the additional binding of a different region of the MHC molecules by the co-receptors (CD4 in the case of T helper cells and CD8 in the case of cytotoxic T cells), together with costimulatory molecules such as CD28, initiates a plethora of signaling cascades within the T cell. These cascades give rise to a complex signaling network, which controls the activation of several transcription factors. These transcription factors, in turn, control the cell's fate, particularly whether the T cell becomes activated and proliferates or not [20]. Therefore, we chose to focus on a limited number of receptors that are known to be central to the decision making process. The high number of kinases, phosphatases, adaptor molecules, and their interactions give rise to a complex interaction network which cannot be interpreted via pure intuition and requires the aid of mathematical tools. Since no sufficient basis of kinetic data is available for setting up a dynamic model of this network, we opted to use logical modeling as a qualitative and discrete modeling framework. Note that there are kinetic models dealing with a smaller part of the network (e.g., [5,21,22]), as well as models of the gene regulatory network governing T cell activation [23].

We recently introduced our approach for the logical modeling of signaling networks [13], and, to exemplify it, we presented a small logical model for T cell activation (40 nodes). However, this model only served to demonstrate applicability and was too incomplete to address realistic complex input–output patterns. In contrast, the model presented herein has been significantly expanded to 94 nodes and refined by a careful reconstruction process (see below). It is thus realistic enough to be verified with diverse experimental data and to test its predictive power.

In this report, the large-scale logical model describing T cell activation and the analysis performed therewith will be presented. First we will show that a number of important structural features can be identified with this model. Then we will show that the model not only reproduces published data on wet lab experiments, but it also predicts non-intuitive and previously unknown responses.

## Results

### Setup of a Curated, Comprehensive Logical Model of T Cell Receptor Signaling

**Figure 1:**
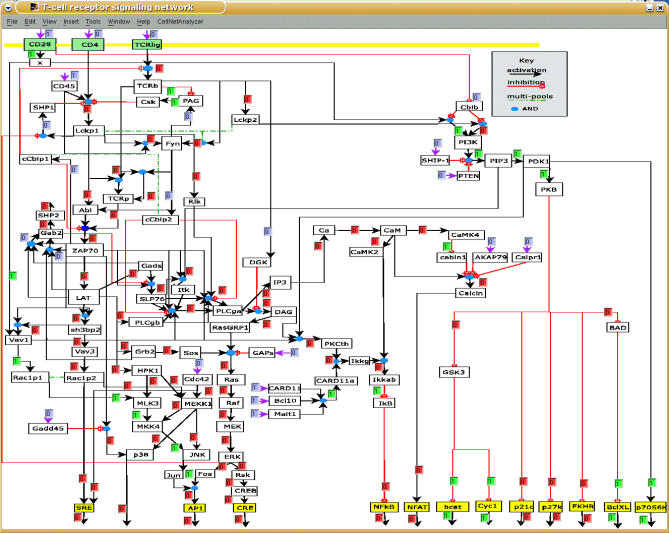
Logical Model of T Cell Activation (Screenshot of CellNetAnalyzer) Each arrow pointing at a species box is a so-called hyperarc representing one possibility to activate that species (see Methods). All the hyperarcs pointing at a particular species box are OR connected. Yellow species boxes denote output elements, while green ones represent (co)receptors. In the shown “early-event” scenario, the feedback loops were switched off, and only the input for the costimulatory molecule CD28 is active (scenario in column 2 of Table 1). The resulting logical steady state was then computed. Small text boxes display the signal flows along the hyperarcs (blue boxes: fixed values prior to computation; green boxes: hyperarcs activating a species (signal flow is 1); red boxes: hyperarcs which are not active (signal flow is 0)).

We have constructed a logical model describing T cell signaling (see Methods and Figure 1), which comprises the main events and elements connecting the TCR, its coreceptors CD4/CD8, and the costimulatory molecule CD28, to the activation of key transcription factors in T cells such as AP-1, NFAT, and NFκB, all of which determine T cell activation and T cell function. In general, the model includes the following signaling steps emerging from the above receptors: the activation of the Src kinases Lck and Fyn, followed by the activation of the Syk-related protein tyrosine kinase ZAP70, and the subsequent assembly of the LAT signalosome, which in turn triggers activation of PLCγ1, calcium cascades, activation of RasGRP, and Grb2/SOS, leading to the activation of MAPKs [20]. Additionally, it includes the activation of the PI3K/PKB pathway that regulates many aspects of cellular activation and differentiation, particularly survival. For the activation of elements that play an important role, but whose regulation is not well-known yet (e.g., Card11, Gadd45), an external input was added. These elements can be considered as points of future extension of the model.

**Figure 2:**
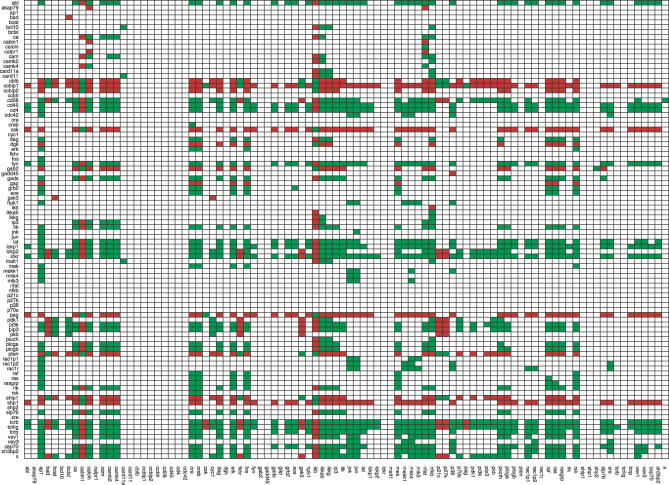
Dependency Matrix of the Logical T Cell Signaling Model (Figure 1) for the Early Events Scenario (τ = 1) The color of a matrix element M_xy_ has the following meaning [13]: (i) dark green: x is a total activator of y; (ii) dark red: x is a total inhibitor of y; (iii) white: no (direct or indirect) influence from x on y.

**Table 1:**
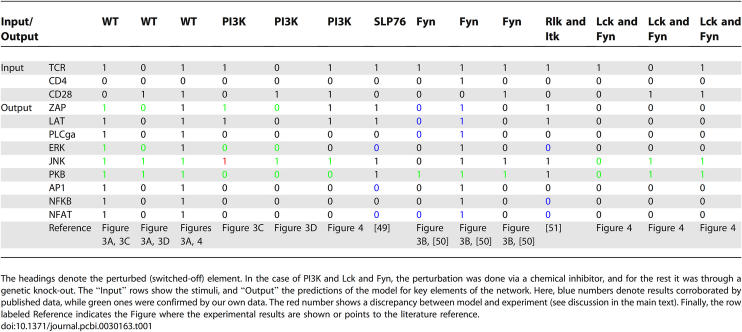
Summary of Predicted Activation Pattern upon Different Stimuli and Knock-Out Conditions

**Figure 3:**
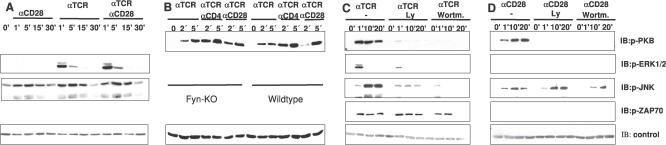
In Vitro Analysis of Model Predictions (A) Activation of ERK and JNK upon CD28, TCR (CD3), or TCR + CD28 stimulation in mouse splenic T cells. (B) Activation of PKB upon TCR, TCR+ CD4, and TCR + CD28 stimulation in Fyn-deficient and heterozygous splenic mouse T cells. (C) Inhibition of PI3K with both Ly294002 and Wortmannin blocks the phosphorylation of PKB, ERK, and JNK, but not ZAP-70 in human T cells. (D) Inhibition of PI3K with both Ly294002 and Wortmannin blocks the phosphorylation of PKB, but not of JNK in human T cells upon CD28 stimulation. As a control, the total amount of ZAP70 (A) or β-actin (B–D) was determined. One representative experiment (of three) is shown.

**Figure 4:**
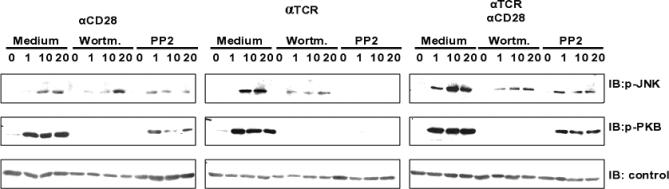
In Vitro Analysis of Src-Kinase Inhibition Inhibition of Src-Kinases (Lck and Fyn) with PP2 blocks TCR-induced but affects only moderately CD28-induced PKB and JNK activation in human T cells; therefore, we concluded that CD28 signaling is not strictly Src-kinase– dependent. The effect was compared with PI3K inhibition via Wortmannin (ccf. Figure 3C and 3D), which blocks the phosphorylation of PKB but not of JNK. β-actin was included as the loading control. One representative experiment (of three) is shown.

As mentioned above, our model, which is documented in a detailed manner in Tables S1 and S2, is based upon *local* interactions (e.g., kinase ZAP70 phosphorylates the adaptor molecule LAT) that are well-established for primary T cells in the literature. We did not use the known *global* information (e.g., stimulation of a receptor leads to the activation of a certain transcription factor) for the model construction. Instead, in simulations, the local interactions give rise to a global behavior which can be compared with available experimental observations (and was thus used to *verify* the model).

Each component in the logical model can be either ON (“1”) or OFF (“0”). We consider a compound to be ON only if it is fully activated and able to trigger downstream events properly; otherwise, it is OFF. Furthermore, we consider two timescales [13]: early (τ = 1) and late (τ = 2), involving processes occurring during or after the first minutes of activation, respectively (the time-scale for each interaction is given in Table S2). Some key regulatory processes such as the degradation of signaling proteins mediated by the E3 ubiquitin ligase c-Cbl [24–26] occur after a certain time, and are thus assigned τ = 2. Therefore, as will be shown later, analysis of signal propagation during the early events reveals which elements become activated, and the consideration of the late events allows a rough approximation to the dynamic behavior (sustained versus transient) of the network.

The model comprises 94 different compounds and 123 interactions that give rise to a complex map of interactions (Figure 1). It is, to the best of our knowledge, the largest Boolean model of a cellular network to date.

### Interaction-Graph-Based Analyses

The first step in our analysis was to examine the interaction graph underlying the logical model. The former can be easily derived from the latter when a special representation of Boolean networks is used (see Methods). The interaction graph is less constrained than the Boolean network since it only captures direct (positive or negative) *effects* of one molecule upon another. Thus, unlike the logical model, the interaction graph cannot describe how different causal effects converging at a certain species are combined. For example, in an interaction graph we may say that A and B have a positive influence on another node C; the logical network is more precise because it expresses that A AND B (or A OR B) are required to activate C. Accordingly, compared with the logical model, an interaction graph requires less a priori knowledge about the network under study which comes at the price that functional predictions are limited. Nevertheless, as demonstrated in this section, a number of important functional features can be revealed from the graph model.

First we studied global properties of the graph. As expected, the graph is connected (i.e., neglecting the arc directions, there is always a path from one node to all others). However, the directed graph contains as a core one strongly connected component with 33 nodes (i.e., for each pair (*a,b*) of nodes taken from this component there is a path from *a* to *b* and from *b* to *a*). This structural organization is related with the bow-tie structure found in other cellular networks (e.g., [7,27]) and implies that the rest of the network (not contained in the strongly connected component) mainly consists in relatively simple input and output layers (including branching cascades) feeding to and from this component.

We continued the interaction-graph-based analysis by computing the feedback loops. Feedback loops are of major importance for the dynamic behavior and functioning of biological networks. Negative feedback loops control homeostatic response and can give rise to oscillations, while positive feedbacks govern multistable behavior (connected to irreversible decision-making and differentiation processes) [15,28–30]. The interaction graph underlying the logical T cell model has 172 feedback loops, 89 thereof being negative. Remarkably, all feedback loops are only active in the second timescale because each loop contains at least one process of the second timescale. The elements of the MAPK cascade are involved in 92% of the feedback loops. This is due to the fact that there is a connection from ERK to the phosphatase SHP1 from the bottom to the top of the network [5]. Due to this connection, the resulting feedback can return to ERK via many different paths, thereby leading to a high number of loops. Indeed, if the ERK → SHP1 connection is not considered, the number of loops is reduced dramatically from 172 to 13 (with only 11 being negative), all located in the upper part of the network. c-Cbl is involved in ∼85% of them, thus underscoring the importance of c-Cbl in the regulation of signaling processes [25,26].

There are 4,538 paths, each connecting one of the three compounds from the input layer (TCR, CD4/CD8, CD28) with one compound in the output layer (transcription factors and other elements controlling T cell activation). The high number of negative paths (2,058) can be traced back to the presence of two negative connections (via DGK and Gab2). In fact, considering the early signaling events within the network, where DGK and Gab2 are not active yet, the number of paths is reduced to 1,530, with only six of them being negative. These paths are from the TCR and CD28 to negative regulators of the cell cycle (p21, p27, and FKHR), having thus a positive effect on T cell proliferation. These and other global effects can be graphically inspected via the dependency matrix [13,31], depicted in Figure 2. Importantly, when considering the timescale τ = 1, there is no ambivalent effect (i.e., via positive *and* negative paths) between any ordered pair (A,B) of species, i.e., A is either a pure activator of B (only positive paths from A to B), or a pure inhibitor of B (only negative paths from A to B), or has no direct or indirect influence on B at all. For example, during early activation, the TCR can only have a positive effect upon AP1 (the array element (TCRb, AP1) in Figure 2 is green). Note that this changes for timescale τ = 2 where, in several cases, a compound influences another species in an ambivalent manner.

### Analysis of the Logical Model

An important aspect that can be studied with a logical model is signal processing and signal propagation and the corresponding response (activation/inactivation) of the nodes upon external stimuli and perturbations (see Methods). One starts the analysis of a scenario by defining a pattern of input stimuli, possibly in combination with a set of nodes that are knocked-out or knocked-in. Then, by an iterative evaluation of the Boolean rules in each node, the signal is propagated through the network, switching each node ON or OFF, respectively (see [13] and Methods). For example, since CD28 (an input) is (permanently) ON in the scenario shown in Figure 1, it will (permanently) activate node X, which will in turn (permanently) activate Vav1, and so forth. In the same scenario, since the input CD4 is OFF, Lckp1 and therefore Abl, ZAP70, and other components cannot become activated and therefore are in the OFF state. In the ideal case, each node can be assigned a uniquely determined state that follows from a given input pattern. In terms of Boolean networks, the set of determined node values then represents a logical steady state. In some cases, in particular when negative feedback loops are active, only a fraction of the elements can be assigned a unique steady state value, whereas other (or even all) nodes might oscillate [15]. However, since in the T cell model all negative feedback loops become active only during timescale τ = 2 (as described above), a complete logical steady state follows for arbitrary input patterns when considering τ = 1.

Using this kind of logical steady state analysis, we first analyzed the activation pattern of key elements upon different stimuli (activation of the TCR and/or CD4 and/or CD28; Table 1) for timescale τ = 1. The model was able to reproduce data from both the literature and our own experiments, providing a holistic and integrated interpretation for a large body of data. The model also predicted a non-obvious signaling event, namely that the activation of the costimulatory molecule CD28 alone leads not only to the activation of PI3K—which is to be expected from a large body of literature dealing with CD28 signaling showing that PI3K binds to the motif YxxM of CD28 [32,33]—but also to the selective activation of JNK, but not ERK. The model predicts a pathway from CD28 to JNK which gives a holistic explanation for this result: the pathway does NOT involve the LAT signalosome, activation of PLCγ1, and Calcium flux, but clearly depends on the activation of the nucleotide exchange factor Vav1 which activates MEKK1 via the small G-protein Rac1 (Figure 1). Clearly, the activating pathway shown in Figure 1 could be identified by a visual inspection of the map (note that we have intentionally drawn the network in such a way that this route can be easily seen). However, in large-scale networks the identification of long-distance pathways by simply following all possible routes becomes infeasible and is particularly complicated if AND connections are involved. Furthermore, since the CD28-induced JNK activation pathway was not expected, one would probably not have searched specifically for this pathway, while the algorithm reveals the whole response of the network.

The prediction made by the model is particularly surprising in light of published data which either suggest that CD28 stimulation alone does NOT activate JNK [34,35] or induces only a weak activation [36]. Driven by this surprising prediction, we performed the corresponding experiments in vitro. As shown in Figure 3A, stimulation of mouse primary T cells with a non-superagonistic CD28 antibody induced an evident and sustained JNK phosphorylation, thus confirming almost perfectly the predicted binary response. Note, the model also predicted that JNK activation does not depend on the activation of PI3K. Again, this prediction was verified by applying a pharmacological inhibitor of PI3K (Figure 3D). The discrepancies with the literature could be due either to the different cellular systems (primary T cells versus T cell lines) or to the different stimulation conditions.

The nature of the kinase involved in CD28-mediated signaling remains unclear. Indeed, application of the Src-kinase inhibitor PP2 that inactivates both Lck + Fyn [37], showed that Src-kinases, which were proposed to mediate CD28 signaling [38], are dispensable for the CD28-mediated activation of JNK (Figure 4). To fit the Src-kinase inhibitor data with the model, it would have been possible to simply bypass the Src-kinase and to draw a causal connection from CD28 to Vav. Such a connection would indeed be justified since it is well established that triggering of CD28 leads to the activation of Vav ([39]; for more details, see Table S2, reactions 35 and 48). However, we preferred to include a to-be-identified kinase X that gets activated by CD28 (Figure 1), in order to keep within the model the information that there is a component to be identified. Potential candidates for kinase X would be members of the Tec-family of PTKs. However, it is difficult to study the signaling properties of these kinases in primary non-transformed cells since specific inhibitors for Tec kinases are not yet available and the corresponding knock-out mice show defects in thymic development. Therefore, as we focused during model generation on well-established data from primary T cells and excluded data obtained from knockout mice showing alterations of thymic development, we did not include it.

The ability of the model to recapitulate the T cell phenotype of a variety of previously described knock-out mice was also tested (Table 1). Indeed, the model could reproduce the phenotype of several knock-outs and again reported a rather unexpected result: activation of the TCR in Fyn-deficient cells selectively triggers the PI3K/PKB pathway. This prediction was subsequently tested using peripheral primary T cells prepared from spleen of Fyn-deficient mice. As shown in Figure 3B, the wet-lab experiments corroborated the model result again.

However, there was an experimental result which the model could not reproduce: TCR-mediated JNK activation is blocked by an inhibitor of PI3K (Figure 3C). In fact, this result is not in accordance with the network because PI3K has no influence upon JNK (see dependency matrix, Figure 2).

To identify potential connections that would explain the experimental data, we applied the concept of Minimal Intervention Sets (MISs; see Methods). A MIS is a irreducible collection of actions (e.g., activation or inactivation of certain compounds), that, if applied, guarantees that a certain goal (a desired behavior) is fulfilled [13]. Here, we computed the MISs by which JNK becomes activated under the experimentally obtained constraint (see Figure 3C) that PI3K is OFF (describing the effect of the PI3K inhibitor), ZAP70 is ON, and that the TCR has been activated. These MISs (Table 2) thus provide a list of minimal combinations of elements that should be directly or indirectly affected by PI3K and thus allow us to explain the observed response of JNK upon inhibiting PI3K. Some of them are obvious, e.g., the first MIS in Table 2 suggests that JNK activation could be directly interacting with PI3K or elements that are located downstream of PI3K (e.g., PIP3). There is currently no convincing experimental evidence for an effect of PI3K on JNK, though. Other MISs in Table 2 suggest that a PI3K-mediated activation of Vav (both 1 and 3 isoforms) is involved, which would be an attractive possibility to explain the experimental data. Indeed, Vav possesses a PH domain which can bind to PIP3, and this mechanism could be important for Vav activation [40], thus making it a reasonable extension of the model.

**Table 2:**
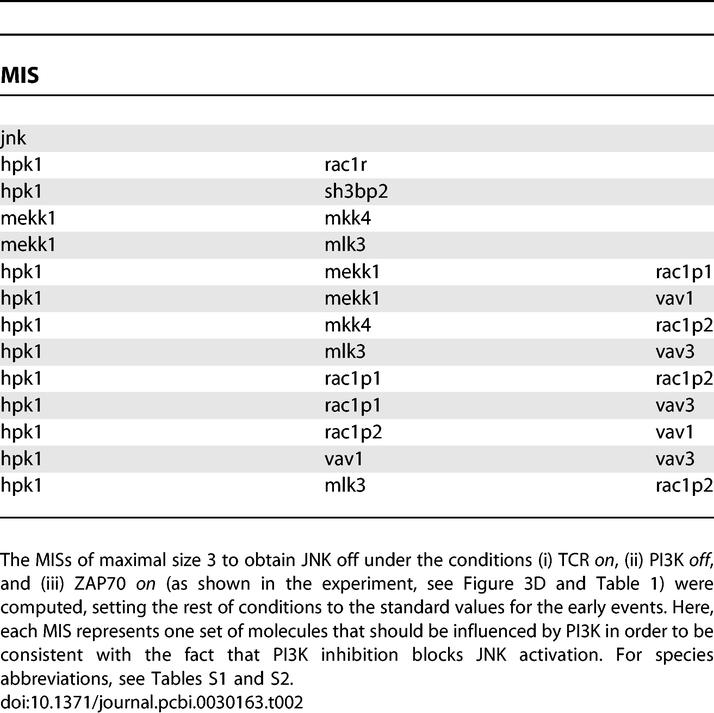
Application of the Minimal Intervention Sets To Identify Candidates To Fill the Gap between PI3K and JNK

Another molecule that could be involved in PI3K-mediated activation of JNK is the serine/threonine kinase HPK1 (see Figure 1 and Tables S1 and S2). Interestingly, HPK1 is phosphorylated by Protein Kinase D1 (PKD1) [41], a kinase whose activation depends on PKC (which in turn is dependent on DAG, downstream of PI3K) for activation. Since the regulation and functional roles of both PKD1 and PKC (with the exception of the θ isoform) are not yet well-established in T cells, we did not include them in the model, but a connection PI3K → PIP3 → Itk → PLCγ → DAG → PKC → PKD1 → HPK1 would be plausible (in which the path from PKC to HPK1 via PKD1 would be new). An alternative could be a Rac-dependent activation of HPK1 [42]; however, this is again a not-well-established connection and thus was not considered.

Definitely, the model requires a direct or indirect connection from PI3K to JNK, and additional experiments are required to assess which of the candidate links predicted by the MISs are relevant in peripheral T cells. This particular example illustrates another useful and important application of our approach: the model not only reveals that a link is missing, but also suggests candidates that can be verified experimentally. Thus, MIS analysis is capable of guiding the experimentalist and helps to plan the corresponding experiments.

As an additional application of MISs, we computed combinations of failures (constitutive activation or inactivation of elements caused for example by mutations) which lead to sustained T cell activation without external stimuli. These failure modes would cause uncontrolled proliferation and thus may be connected to diseases such as leukemia or autoimmunity. Interestingly, components occurring in the MISs with few elements (Table 3) are in fact known oncogenes: ZAP70 [43], PI3K [44], Gab2 [45], and PLCγ1 [46] (and SLP76 is directly involved in PLCγ1 activation).

**Table 3:**
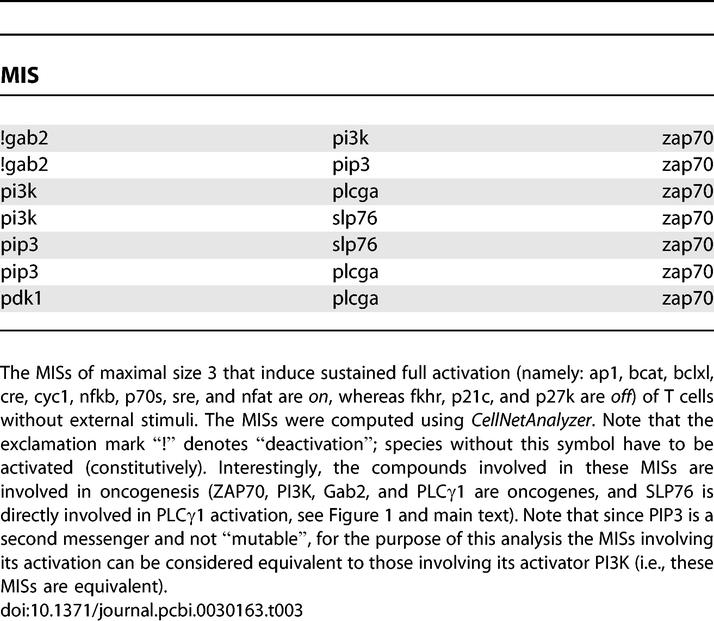
Minimal Intervention Sets To Produce the Full Activation Pattern in T Cells

### Robustness and Sensitivity Analysis of the Logical Model

Strongly related to the idea of MISs is a systematic evaluation of the network response if the model is confronted with failures. By considering a failure as something that happens to the cell by an internal or external event (e.g., a mutation), we may assess the *robustness*—one of the most important properties of living systems [47]—of the network. In contrast, if we consider the failure as an error that has been introduced during the modeling process (due to incomplete knowledge), then we are assessing the *sensitivity* of the model with respect to the predictions it makes. Accordingly, to study robustness and sensitivity issues, we (i) removed systematically each single interaction from the network, (ii) recomputed the scenarios given in Table 1, and (iii) compared the new predictions with the 126 original predictions (Table 1), ranking the interactions according to the number of introduced changes produced (Table 4). As an average value, 4.76 errors were introduced per simulated failure, which corresponds to 3.78% of the total numbers of predictions. The most sensitive interactions are mainly located in the upper part of the network and activate components such as the T cell receptor (TCRb), ZAP70, LAT, Fyn, or Abl. It is intuitively clear that the network is very sensitive to failures (again, caused either by internal/external events or modeling errors) in these upper nodes because all pathways branching downstream are governed by them. Accordingly, the validation of our model (with the data from Table 1) is most sensitive to modeling errors in the upper part of the network. We also note that species that can be activated by more than one interaction (e.g., PI3K) are significantly less sensitive to single interaction failures since alternative pathways exist. Regarding robustness, it is worth emphasizing that in the worst case about 30% of the original predictions are affected after removal of an interaction, indicating that there is no “all-or-nothing” interaction in the network.

**Table 4:**
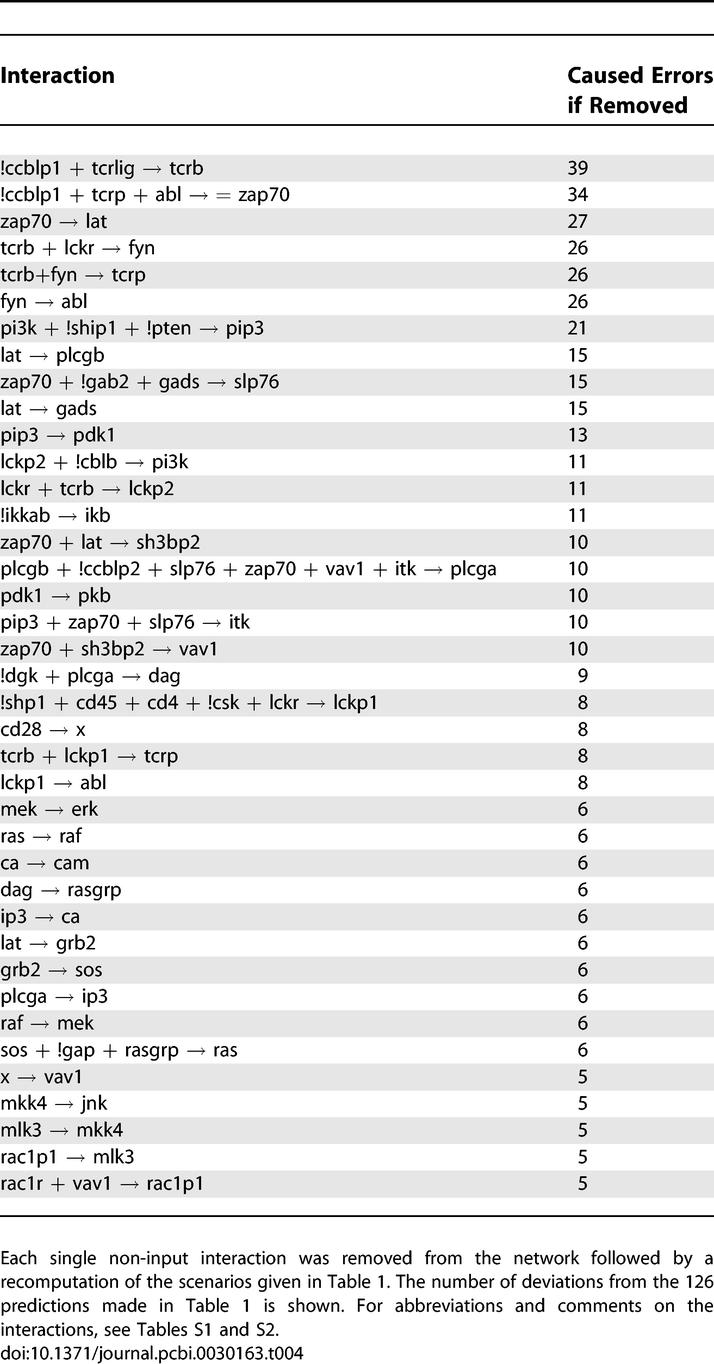
Robustness Analysis: Ranked List of the Most Sensitive Interactions

We have also performed the same analysis for the removal of a species (instead of an interaction) which basically led to the same results (unpublished data). However, the removal of a node can be seen as a stronger intervention in the network than deleting an interaction, as the former simulates the simultaneous removal of all interactions pointing at that species. Accordingly, deleting nodes implies some stronger deviations from the original predictions.

### Qualitative Description of the Dynamics

So far we have analyzed which elements within the signaling network get activated upon signal triggering (i.e., for the first timescale τ = 1). This is due to the fact that a large corpus of data for these conditions is available (see Table 1). However, it is important to note that the model is also able to roughly predict the dynamics upon different stimuli and conditions.

**Figure 5:**
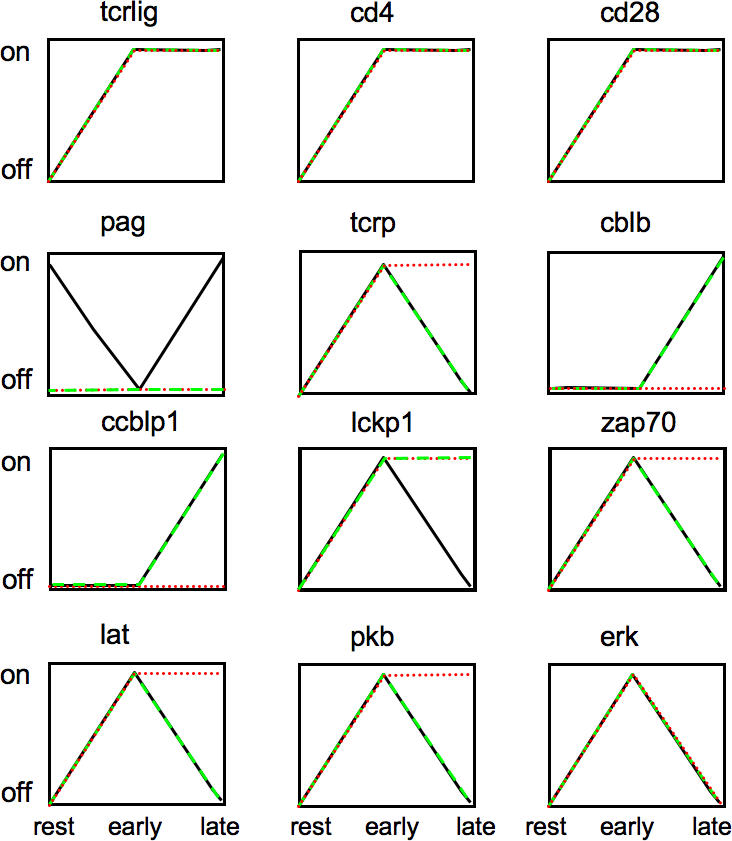
Considering Different Time Scales, a Rough Description of the Dynamics Can Be Obtained The activation of key elements upon activation of the TCR, the coreceptor CD4, and the costimulatory molecule CD28 is represented at the resting state, τ = 0 (no inputs); early events τ = 1 (input(s), no feedback loops); and later-time events, τ = 2 (input(s), feedback loops). The black lines correspond to a wild type while the green ones to a PAG KO. Note that the absence of PAG has no effect on key downstream elements of the cascade, due to the redundant role of other negative regulatory mechanisms (degradation via c-Cbl and Cbl-b, Gab-2 mediated inhibition of PLCγ1). Multiple knock-out of these regulatory molecules leads to sustained activation of key elements (red lines).

The modus operandi goes as follows: first, one computes the steady state values with no external input (τ = 0). Subsequently, the steady state for τ = 1 is computed as described above. Finally, one computes the state of the “slow” interactions (those only active at τ = 2) as a function of the values at τ = 1, and subsequently recomputes the steady states. This provides the response at late events, τ = 2. The results obtained can be plotted in a time-dependent manner (Figure 5). Here, one can also investigate the effect of different knock-outs. For example, the absence of PAG has no effect on key downstream elements of the cascade, due to the redundant role of other negative regulatory mechanisms (specifically, the degradation via c-Cbl and Cbl-b, and Gab-2–mediated inhibition of PLCγ1). Only a multiple knock-out of these regulatory molecules leads to sustained activation of key elements. Thus, these results point to a certain degree of redundancy in negative feedbacks for switching off signaling.

This sort of qualitative analysis of the dynamics shows the ability of the Boolean approach to reproduce the key dynamic properties (transient versus sustained) of a signaling process.

## Discussion

In this contribution, a logical model describing a large signaling network was established and analyzed. We set up a comprehensive Boolean model describing T cell signaling and performed logical steady state analyses unraveling the processing of signals and the global input–output behavior. Moreover, by converting the logical model into an interaction graph, we extracted further important features, such as feedback loops, signaling paths, and network-wide interdependencies. The latter can be captured in a dependency matrix (as in Figure 2) which provides thousands of qualitative predictions that can be falsified in perturbation experiments. The logical model reproduces the global behavior of this complex network for both natural and perturbed conditions (knock-outs, inhibitors, mutations, etc.). Its validity has been proven by reproducing published data and by predicting unexpected results that were then verified experimentally. Table 1 summarizes the results of 14 different scenarios, in which the logical model predicted 126 states. For 44 of them, experimental data was available (15 from literature and 29 from our own experiments) confirming the predictions, except in the case discussed above.

Furthermore, we clearly show that the concept of intervention sets allows one (a) to identify missing links in the network, (b) to reveal failure modes that can explain the effects of a physiological dysfunction or disease, and (c) to search for suitable intervention strategies, while keeping track of potential side effects, which is valuable for drug target identification.

Compared with a kinetic model based on differential equations, a Boolean approach is certainly limited regarding the analysis of quantitative and dynamical aspects, and it certainly cannot answer the same questions. However, to establish such a model requires mainly the topology and only a relatively small amount of quantitative data; hence, a combination of information which is currently available in large-scale networks. Although the model itself is qualitative (i.e., discrete), it enables us not only to study qualitative aspects of signaling networks, but it can also be validated by semi-quantitative measurements such as those in Figures 3 and 4. In summary, with the network involved in T cell activation as a case study, our approach proved to be a promising in silico tool for the analysis of a large signaling network, and we think that it holds valuable potential in foreseeing the effects of drugs and network modifications. Although sometimes the results of a logical model may (afterward) appear to be obvious (as in the case of the CD28-mediated JNK connection), it enables an exhaustive and rigorous analysis of the information processing taking place within a signaling network. Such a systematic analysis becomes infeasible for a human being in large-scale systems. In addition, the LIH can represent the situation of varying cofactor functions; for example, that two substances A AND B are required to activate a third substance C, but activation of C in the presence of A and a fourth substance D requires B not to be present.

Certainly, the logical model for T cell activation is far from complete. We are just at the beginning of the reconstruction process and other receptors and their pathways need to be included. However, we feel that already in its current state, the model may prove useful to inspire immunologists to ask new questions which may first be answered in silico. Furthermore, the model may also provide a framework for those who may endeavor to quantitatively model TCR signaling.

## Methods

### Logical network representation and analysis.

We began construction of the signaling network for primary T cells by collecting data from the literature and from our own experiments providing well-established connections (Tables S1 and S2). As a first (intermediate) result, we obtain an interaction graph. Interaction graphs are signed directed graphs with the molecules (such as receptor, phosphatase, or transcription factor) as nodes and signed arcs denoting the direct influence of one species upon another, which can either be activating (+) or inhibiting (−). For example, a positive arc leads from MEK to ERK because the first phosphorylates and thereby activates the second (Figure 1). From the incidence matrix of an interaction graph we can identify important features such as feedback loops as well as signaling paths and network-wide interdependencies between pairs of species (e.g., perturbing A may have no effect on B as there is no path connecting A to B). Algorithms related to these analyses are well-known [48] and were recently presented in the context of signaling networks [13]. However, from interaction graphs we cannot conclude which combinations of signals reaching a species along the arcs are required to activate that species. For example, in Figure 1, Jun AND Fos are required to form active AP1.

For a refined representation of such relationships, we use a logical (or Boolean) model in which we introduce discrete states for the species (here the simplest (binary) case: 0 = inactive or not present; 1 = active or present) and assign to each species a Boolean function. Here we use a special representation of Boolean functions known as disjunctive normal form (DNF, also called “sum of product” representation) which uses exclusively AND, OR, and NOT operators. A Boolean network with Boolean functions in disjunctive normal form can be intuitively drawn and stored as a hypergraph (LIH) [13], which is well-suited for studying the information flows and input–output relationships in signal transduction networks (Figure 1). In this hypergraph, each hyperarc connects its start nodes with an AND operation (indicated by a blue circle in Figure 1) and each hyperarc represents one possibility for how its end node can be activated or produced (note that hyperacs may also have only one start node, i.e., they are then “graph-like” arcs). Red branches indicate species that enter the hyperarc with their negated value. For example, PLCγ-1 (PLCga in Figure 1) AND NOT DGK activates DAG (see Figure 1). Note that each LIH has a unique underlying interaction graph (which can be easily derived from the LIH representation by splitting the AND connections), whereas the opposite is, in general, not true.

Within this logical framework we may study the effect of a set of input stimuli (typically ligands) on downstream signaling by computing the logical steady state [13] that results by propagating the signals through the network from the input to the output layer. It seems worthwhile to remark that the updating assumption (synchronous versus asynchronous [14,15])—which must usually be made when dealing with dynamic Boolean networks—is not relevant here as we focus on the logical steady states, which are equivalent in both cases. Sometimes a logical steady state is not unique or does not exist due to the presence of feedbacks loops. However, many feedback loops become active only in a longer timescale justifying setting them OFF in the first wave of signal propagation (allowing them to be switched ON for the second timescale). This has been used here for several feedback loops (see main text and Table S2). The effect of knocking-out a species can be tested by re-computing the (new) logical steady state for the respective stimuli. MISs satisfying a given intervention goal can be computed by systematically testing sets of permanently activated or/and deactivated nodes [13,31].

All mathematical analyses and computations have been performed with our software tool *CellNetAnalyzer* [31], a comprehensive user interface for structural analysis of cellular networks. *CellNetAnalyzer* and the T cell model can be downloaded for free (for academic use) from http://www.mpi-magdeburg.mpg.de/projects/cna/cna.html.

### Immunoblotting.

Human or mouse T cells were purified using an AutoMACS magnetic isolation system according to the manufacturer's instructions (Miltenyi, http://www.miltenyibiotec.com). Mouse T cells were stimulated with 10 μg/ml of biotinylated CD3ɛ (a subunit of the TCR) antibody (145–2C11, BD Biosciences, http://www.bdbiosciences.com/), 10 μg/ml of biotinylated CD28 antibody (37.51, BD Biosciences), CD3 plus CD28 mAbs, or with CD3 plus 10 μg/ml of biotinylated CD4 (GK1.5, BD Biosciences) followed by crosslinking with 25 μg/ml of streptavidin (Dianova, http://www.dianova.de) at 37 °C for the indicated periods of time. Human T cells were stimulated with CD3ɛ mAb MEM92 (IgM, kindly provided by Dr. V. Horejsi, Prague, Czech Republic) or with CD3 plus CD28 mAbs (248.23.2). Cells were lysed in buffer containing 1% NP-40, 1% laurylmaltoside (N-dodecyl β-D-maltoside), 50 mM Tris pH 7.5, 140 mM NaCl, 10mM EDTA, 10 mM NaF, 1 mM PMSF, 1 mM Na_3_VO_4_. Proteins were separated by SDS/PAGE, transferred onto membranes, and blotted with the following antibodies: anti-phosphotyrosine (4G10), anti-ERK1/2 (pT202/pT204), anti-JNK (pT183/pY185), anti-phospho-Akt (S473) (all from Cell Signaling, http://www.cellsignal.com/), anti-ZAP70 (pTyr 319, Cell Signaling), anti-ZAP70 (cloneZ24820, Transduction Laboratories, http://www.bdbiosciences.com/), or against β-Actin (Sigma, http://www.sigmaaldrich.com/). Where PI3K and src-kinase inhibitors were used, T cells were treated with 100 nM Wortmannin (Calbiochem, http://www.emdbiosciences.com) or 10 μM PP2 (Calbiochem) for 30 min at 37 °C prior to stimulation. All experiments have been repeated three times and reproduced the shown results.

## Supporting Information

Table S1 List of Compounds in the Logical T Cell Model Model name corresponds to the name in Figure 1 and Table S2. Common abbreviations are those usually used in the literature, while name is the whole name. Type classifies the molecules, if applies, as follows: K = Kinase, T = Transcription Factor, P = Phosphatase, A = Adaptor Protein, R = Receptor, G = GTP-ase. In the case where two pools of a molecule were considered, a “reservoir” was included which was required for both pools. This allows us to perform a simultaneous knock-out of both pools.
(56 KB PDF)

Table S2 Hyperarcs of the Logical T Cell Signaling Model (see Figure 1 and Methods) Exclamation mark (“!”) denotes a logical NOT, and dots within the equations indicate AND operations. The names of the substances in the explanations are those used in the model and Figure 1; the biological names are displayed in Table S1. In the case where two pools of a molecule were considered (e.g., lckp1 and lckp2), a “reservoir” (lckr) was included which was required for both pools. This allows us to perform a simultaneous knock-out of both pools acting on the reservoir.
(183 KB PDF)

## Abbreviations

LIH: logical interaction hypergraph
MHC: Major Histocompatibility Complex
MIS: Minimal intervention set
TCR: T cell receptor
