# Supplementary material for: A Logical Model Provides Insights into T Cell Receptor Signaling
Source: PLoS Comput Biol. 2007 Aug 24;3(8):e163. doi: 10.1371/journal.pcbi.0030163 (PMC1950951; doi:10.1371/journal.pcbi.0030163)
Supplement: Supplementary file 1 [file pcbi.0030163.st001.pdf]

Supplementary Table 1. List of compounds in the logical T-cell model. Model name corresponds to the name in Fig. 1 and the Supplementary Table 2. Common abbreviations are those usually used in the literature, while name is the whole name. Type classifies the molecules, if applies, as follows: K =Kinase, T=Transcription Factor, P = Phosphatase, A =Adaptor Protein, R = Receptor, G= GTP-ase. In the case where two pools of a molecule were considered, a'reservoir' was included which was required for both pools. This allows to perform a simultaneous knock-out of both pools.

Nr	Model Name	Common abbreviation	Name	Type
1	abl	Abl	Abelson protein tyrosine kinase	K
2	akap79	Akap79	A kinase (PRKA) anchor protein 79	A
3	ap1	Ap1	v-jun sarcoma virus 17 oncogene homology	T
4	bad	Bad	BCL2-antagonist of cell death	
5	Bcat	-catenin	Catenin (cadherin-associated protein) beta 1	
6	bcl10	BCL10	B-cell CLL/lymphoma 10	
7	bclxl	BCL2L1	BCL2-like 1	
8	ca	CA, Ca2+	Calcium	
9	cabin1	Cabin1, CAIN	Calcineurin-binding protein	
10	calcin	Calcineurin	Calcineurin	P
11	calpr1	Csp1	Calcipressin 1	
12	cam	CaM	Calmodulin	
13	camk2	Camk2,CamKII	calcium/calmodulin-dependent protein kinase (CaM kinase) II	K
14	camk4	CamK4, CamKIV	calcium/calmodulin-dependent protein kinase IV	K
15	card11a	CARD11 + CARMA1 + BCL10	Complex of CARD11, CARMA1, and BCL10, which is their fully active form (see Fig. 1)	
16	card11	CARMA1, CARD11	caspase recruitment domain family, member 11	
17	cblb	Cbl-b	Cas-Br-M (murine) ecotropic retroviral transforming sequence b	
18	ccblp1	c-Cbl	Casitase B-lineage lymphoma proto-oncogene (ZAP70-dependent pool)	A
19	ccblp2	c-Cbl	Casitase B-lineage lymphoma proto-oncogene (Fyn-dependent pool)	A

Nr	Model Name	Common abbreviation	Name	Type
20	ccblr	c-Cbl	Casitase B-lineage lymphoma proto-oncogene (Reservoir, see legend)	A
21	cd28	CD28	CD28 antigen (Tp44)	R
22	cd45	CD45, PTPRC	protein tyrosine phosphatase, receptor type, C	P
23	cd4	CD4, p55	CD4 antigen (p55)	R
24	cdc42	CDC42, BB1	cell division cycle 42 (GTP binding protein, 25kDa)	G
25	cre	Cre	cAMP responsive element binding protein 1	
26	creb	CREB, CREB1	cAMP responsive element	T
27	csk	Csk	c-src tyrosine kinase	K
28	cyc1	Cyc1	cytochrome c-1	
29	dag	DAG	Diacylglycerol	
30	dgk	DGK	diacylglycerol kinase	K
31	erk	ERK, MAPK1	Extracellular signal-regulated kinases, mitogen-activated protein kinase 1	K
32	fkhr	FOXO1, fkhr	forkhead box O1A	T
33	fos	Fos, FRA	FOS-like antigen	
34	fyn	Fyn	FYN oncogene related to SRC, FGR, YES	K
35	gab2	Gab2	GRB2-associated binding protein 2	A
36	gadd45	GADD45A	growth arrest and DNA-damage-inducible, alpha	
37	gads	GRAP-2, GADS, P38	GRB2-related adaptor protein 2	A
38	gap	GAP	GTPase-activating protein	
39	grb2	Grb2	growth factor receptor-bound protein 2	
40	sos	Sos1	Son of sevenless homolog 1	
41	gsk3	GSK3	glycogen synthase kinase 3	K
42	hpk1	HPK1, MAP4K1	Hematopoietic progenitor kinase 1, mitogen-activated protein kinase kinase kinase kinase 1	K
43	ikb	IκB	Inhibitor protein IκB	
44	ikkab	IκKA + IκKB	inhibitor of kappa light polypeptide gene enhancer in B-cells, kinase alpha and kinase beta	

Nr	Model Name	Common abbreviation	Name	Type
45	ikkg	IκKG	inhibitor of kappa light polypeptide gene enhancer in B-cells, kinase gamma	
46	ip3	IP3K	inositol 1,4,5-trisphosphate	K
47	itk	ITK	IL2-inducible T-cell kinase	K
48	jnk	JNK, SAPK, MAPK8	mitogen-activated protein kinase 8	K
49	jun	JUN, ap1	v-jun sarcoma virus 17 oncogene homolog	T
50	lat	LAT	linker for activation of T cells	A
51	lckp1	Lck	lymphocyte-specific protein tyrosine kinase (CD4-dependent pool)	K
52	lckp2	Lck	lymphocyte-specific protein tyrosine kinase (CD28/TCR-dependent pool)	K
53	lckr	Lck	lymphocyte-specific protein tyrosine kinase (Reservoir, see legend)	K
54	malt1	MALT1	mucosa associated lymphoid tissue lymphoma translocation gene 1	
55	mek	MEK, MAP2K, MAPKK	mitogen-activated protein kinase kinase	K
56	mekk1	MEKK1, MAP3K1, MAPKKK1	mitogen-activated protein kinase kinase kinase	K
57	mkk4	MKK4, MAP2K4, MAPKK4	mitogen-activated protein kinase kinase 4	K
58	mlk3	MLK3, MAP3K11	mitogen-activated protein kinase kinase kinase 11	K
59	nfat	NFATC1, NFATC2, NFATC3	nuclear factor of activated T-cells, cytoplasmic, calcineurin-dependent 1	T
60	nfkb	NfκB	nuclear factor of kappa light polypeptide gene enhancer in B-cells 1, 2	T
61	p21c	P21Cip1, Cdkn1a, p21	cyclin-dependent kinase inhibitor 1A (p21, Cip1)	
62	p27k	CDKN1B, p27Kip1	cyclin-dependent kinase inhibitor 1B (p27, Kip1)	
63	p38	P38, MAPK14	mitogen-activated protein kinase 14	K
64	p70s	RPS6KB1, p70s6k	ribosomal protein S6 kinase, 70kDa, polypeptide 1	K

Nr	Model Name	Common abbreviation	Name	Type
65	pag	PAG1	phosphoprotein associated with glycosphingolipid microdomains 1	A
66	pdk1	PDPK1, PDK1	3-phosphoinositide dependent protein kinase-1	K
67	pi3k	PI3K, PIK3CA	phosphoinositide-3-kinase, catalytic, alpha polypeptide	K
68	pip3	PIP3	Phosphatidylinositol (3,4,5)-trisphosphate	
69	pkb	PKB, AKT1	v-akt murine thymoma viral oncogene homolog 1	K
70	pkcth	Prkcq, PKC-γ	protein kinase C, theta	K
71	plcga	Active form of PLCγ1, PLC-gamma-1	phospholipase C, gamma 1	
72	plcgb	PLCG1, PLC-γ-1 bound to LAT (but not active, see plcga)	phospholipase C, gamma 1	
73	pten	PTEN	phosphatase and tensin homolog (mutated in multiple advanced cancers 1)	P
74	rac1p1	RAC1, p21-Rac1, MIG5	ras-related C3 botulinum toxin substrate 1 (rho family, small GTP binding protein Rac1) (Vav-1 dependent pool)	G
75	rac1p2	RAC1, p21-Rac1, MIG5	ras-related C3 botulinum toxin substrate 1 (rho family, small GTP binding protein Rac1) (Vav-3 dependent pool)	G
76	rac1r	RAC1, p21-Rac1, MIG5	ras-related C3 botulinum toxin substrate 1 (rho family, small GTP binding protein Rac1) (Reservoir, see footnote)	G
77	raf	RAF1	v-raf-1 murine leukemia viral oncogene homolog 1	K
78	ras	Ras	Ras	G
79	rasgrp	RasGRP1	Ras guanyl releasing protein 1	
80	rlk	TXK, RLK, TKL	TXK tyrosine kinase	K
81	rsk	RPS6KA1, RSK	ribosomal protein S6 kinase, 90kDa, polypeptide 1	K

Nr	Model Name	Common abbreviation	Name	Type
82	Sh3bp2	3BP2, SH3BP2	SH3-domain binding protein 2	A
83	ship1	INPP5D, SHIP1	inositol polyphosphate-5-phosphatase, 145kDa	P
84	shp1	Shp1, PTPN6	protein tyrosine phosphatase, non-receptor type 6	P
85	shp2	SHP2, PTPN11	protein tyrosine phosphatase, non-receptor type 11 (Noonan syndrome 1)	P
86	slp76	LCP2, SLP76	lymphocyte cytosolic protein 2 (SH2 domain containing leukocyte protein of 76kDa)	A
87	sre	Sre	Serum responsive element	
88	tcrb	TCR	T-cell Receptor (bound to ligand)	R
89	tcrlig	PMHC, Ab	Ligand of T cell receptor (peptide-MHC complex or antibody)	R
90	tcrp	TCR	T-cell Receptor phosphorylated	R
91	vav1	VAV1	Vav 1 oncogene	
92	vav3	VAV3	Vav 3 oncogene	
93	X	-	Non-identified kinase involved in CD28-mediated signaling (see main text)	K
94	zap70	ZAP70	zeta-chain (TCR) associated protein kinase 70kDa	K
