# Supplementary material for: A Logical Model Provides Insights into T Cell Receptor Signaling
Source: PLoS Comput Biol. 2007 Aug 24;3(8):e163. doi: 10.1371/journal.pcbi.0030163 (PMC1950951; doi:10.1371/journal.pcbi.0030163)
Supplement: Supplementary file 2 [file pcbi.0030163.st002.pdf]

Supplementary Table 2. Hyperarcs of the logical T-cell signaling model (see Fig. 1 and methods). Exclamation mark ('!') denotes a logical NOT and dots within the equations indicate AND operations. The names of the substances in the explanations are those used in the model and Fig. 1; the biological names are displayed in the Supplementary Table 1. In the case where two pools of a molecule were considered (e.g. lckp1 and lckp2), a'reservoir' (lckr) was included which was required for both pools. This allows to perform a simultaneous knock-out of both pools acting on the reservoir.

Nr	Reaction	τ	Documentation
1	$\rightarrow$ cd28	1	Binding of ligand or antibody to cd28 is an input of the model.
2	$\rightarrow$ cd4	1	Binding of ligand or antibody to cd4 is an input of the model.
3	$\rightarrow$ tcrlig	1	Binding of ligand or antibody to the tcr is an input of the model.
4	!bad $\rightarrow$ bclxl	1	bad inhibits bclxl[1, 2].
5	!cabin1 · !calpr1 · !akap79 · cam $\rightarrow$ calcin	1	cam binds to and activates calcineurin (calcin), while cabin1, calpr1, akap79 inhibit calcin[3, 4, 5].
6	!camk4 $\rightarrow$ cabin1	1	camk4 regulates via phosphorylation nuclear export of Cabin1[6].
7	card11a · pkcth $\rightarrow$ ikkg	1	The complex card11+bcl10 +malt1 is required for ikkg activation [7, 8, 9]. Phosphorylation, probably via pkcth[10], is also required.
8	!ccblp1 · tcrlig $\rightarrow$ tcrb	1	Binding of ligand activates the tcr, while active ccbl ubiquinates it, thus leading to tcr degradation[11].
9	!ccblp1 · tcrp · abl $\rightarrow$ zap70	1	abl phosphorylates and thus activates zap70[12] once it is bound to the tcr. Active ccbl can degrade zap70.
10	!cd28 $\rightarrow$ cblb	2	cd28 induces cblb ubiquitination and degradation[13] after the early events thus, τ=2.
11	!dgk · plcga $\rightarrow$ dag	1	The active form of plc γ 1 (plcga) splits pip2 into diacylglycerol (dag) and ip3 (see hyperarc 83)[11]. Active dgks degrade dag into phosphatic acid[14].
12	!erk · lckp1 $\rightarrow$ shp1	2	lck phosphorylates shp1 leading to its activation which allows it to dephosphorylate and thus deactivate lck. erk phosphorylates lck at p59, protecting it from shp1's effect[15, 16]. Since shp1 activation comes some time after lck activation, it takes place at τ=2.

Nr	Reaction	τ	Documentation
13	!gab2 · zap70 · gads → slp76	1	slp76 associates with lat via gads[17, 18]. gab2 competes for binding, and thus inhibits binding of slp76 to gads[19, 20].
14	!gsk3 → bcat	1	Gsk3 inhibits bcat[21].
15	!gsk3 → cyc1	1	Gsk3 inhibits cyc1[21].
16	!ikb → nfkb	1	nfkb is retained in the cytoplasm by tight binding to the inhibitory protein ikb[11].
17	!ikkab → ikb	1	ikb is phosphorylated by ikkab, leading to its ubiquination and subsequent degradation[11, 22].
18	ikkg · camk2 → ikkab	1	Both the regulatory molecule ikkg and phosphorylation probably (but not only) via camk2 are required for the activation of the kinase subunits ikkalpha and beta (ikkab)[7, 8, 9].
19	!pkb → bad	1	pkb inhibits bad[23].
20	!pkb → fkhr	1	pkb inhibits fkhr[23].
21	!pkb → gsk3	1	pkb inhibits gsk3 [21, 23].
22	!pkb → p21c	1	pkb inhibits p21c [21, 23].
23	!pkb → p27k	1	pkb inhibits p27k [21, 23].
24	!gadd45 · zap70 → p38	1	gadd45 inhibits the zap70 mediated activation of p38[24].
25	!shp1 · cd45 · cd4 · !csk · lckr → lckp1	1	Full activation of the cd4-bound pool (there is also a tcr-dependent pool, see hyperarc 62/64 and legend) of lck requires dephosphorylation of the negative regulatory site (by cd45, and in absence of csk, which phosphorylates it) and autophosphorylation of the positive regulatory site, which cd4-bound lck can perform upon cd4 crosslinking[25].
26	!tcrb → pag	1	upon ligand binding to the tcr, pag is dephosphorylated by an unidentified phosphatase (probably cd45)[26].
27	ap1 →	1	the transcription factor ap1 is an output of the model.
28	bcat →	1	bcat is an output of the model.
29	bclxl →	1	bclx is an output of the model.
30	ca → cam	1	calcium binds to calmodulin and this complex to calcineurin[27].
31	calcin → nfat	1	calcineurin dephosphorylates nfat leading to nuclear translocation and activation of nfat[11, 28, 22].
32	cam → camk4	1	camk2 activation is dependent on calmodulin (cam)[29].

Nr	Reaction	τ	Documentation
33	ccblr · fyn ⟶ ccblp2	2	Upon Fyn phosphorylation, ccbl can inhibit plcg[30]. This is one out of 2 mechanisms ccbl is involved in, and we call it ccblp2 (pool 2, see legend). Since ccbl mediated inhibition is slower than the early events, τ=2.
34	ccblr · zap70 ⟶ ccblp1	2	ccbl binds to activated (and thus phosphorylated) zap70, leading to the ubiquination and subsequent degradation of zap70 and tcr[31]. This is one out of 2 mechanisms ccbl is involved in, and we call it ccblp1 (pool 1, see legend). Since ccbl mediated degradation has to be slower than the early events, τ=2.
35	x ⟶ vav1	1	CD28 stimulation leads to Vav1 activation[32, 33, 34, 35], a process mediated by a yet unidentified kinase (see hyperarc 48).
36	cdc42 ⟶ mekk1	1	The GTP bound cdc42 (and rac1, see hyperarc 87) is able to bind mekk1[36]; CD28 activates mekk1 in a cdc42 mediated manner[37].
37	cre ⟶	1	cre is an output of the model.
38	creb ⟶ cre	1	The creb protein is a transcription factor that binds to cre ativating the related genes[22].
39	cyc1 ⟶	1	cyc1 is an output of the model, and is involved in cell cycle regulation[22].
40	dag ⟶ rasgrp	1	dag causes the cytoplasmic rasgrp1 to move to the golgi, where it can act on Golgi associated-ras[38, 39]. Even though pkcth phosphorylates rasgrp at t184,[40] we did not include connection pkcth ⟶ rasgrp1 since this effect is not specific to pkcth, but general to other pkcs (less well-characterized in T cells and therefore not included in the model); inclusion of this effect would make this step strictly dependent on pkcth, which is not the case.
41	dag · vav1 · pdk1 ⟶ pkcth	1	Activation of pkcth requires binding to dag, phosphorylation by pdk1[41], and vav1[42].
42	erk ⟶ fos	1	erk phosphorylates fos[11].
43	erk ⟶ rsk	1	erk activates rsk via phosphorylation[43].
44	fkhr ⟶	1	The transcription factor fkhr is an output of the model.

Nr	Reaction	τ	Documentation
45	fos · jun → ap1	1	Binding of jun with fos leads to the formation of ap1[11, 22].
46	fyn → abl	1	abl kinases are activated following tcr stimulation via a Src kinase (lck or fyn, see hyperarc 59)[12].
47	fyn → pag	2	fyn phosphorylates pag[26], leading to the binding of csk. This process takes place 3-5 min after tcr activation, and thus it belongs to the time scale τ=2[44].
48	cd28 → x	1	Vav1 activation requires cd28 activation[32, 33, 35] and is mediated by an non-identified kinase x (see hyperarcs 35 and 63).
49	gab2 → shp2	1	Gab2 recruits shp2[45].
50	gads · lat · zap70 → gab2	2	zap70 phosphorylates gab2 upon binding to lat and gads[19, 20]. This process must take place after the early events to allow signal propagation, thus τ=2.
51	grb2 · lat · zap70 → gab2	2	zap70 phosphorylates gab2 upon binding to lat and grb2[19, 20]. This process must take place after the early events to allow signal propagation, thus τ=2.
52	hpk1 → mekk1	1	hpk1 binds and phosphorylates mekk1[46].
53	hpk1 → mlk3	1	hpk1 binds and phosphorylates mlk3[47].
54	ip3 → ca	1	Binding of ip3 to the ip3 receptor in the endoplasmatic reticulum leads to the release of calcium[48].
55	jnk → jun	1	jnk phosphorylates jun[22].
56	lat → grb2	1	grb2 (which in turn binds sos) can bind to phosphorylated lat[49][17].
57	lat → hpk1	1	hpk1 binds to lat and is recruited to the lipid raftss[50].
58	lat → plcgb	1	plcgamma binds to lat[17, 18].
59	lckp1 → abl	1	abl kinases are activated following tcr stimulation via a Src kinase (lck or fyn, see hyperarc 46)[12].
60	lckp1 → rlk	1	lck phosphorylates rlk leading to its activation[51].
61	lckp1 · cd45 → fyn	1	lck activates fyn[52], a process where the dephosphorylation of the negative regulatory site of fyn by cd45 is also required.
62	lckp2 · !cblb → pi3k	1	pi3k is dependent on the Src kinase lck for activation[53]. Additionally, cblb promotes pi3k ubiquination[54].

Nr	Reaction	τ	Documentation
63	x · !cblb → pi3k	1	pi3k is also activated upon CD28[55, 56] via an non-determined kinase x (see hyperarc 48). Even though Lck has been proposed to be involved in this process[57, 58, 59, 60], our experiments show that, at least for primary human T-cells, PI3K activation is not strictly Src-kinase dependent (see Fig. S4). A reasonable candidate would be a Tec kinase, but since it is not experimentally verified, we keep an undetermined x.
64	lckr · tcrb → lckp2	1	The activation of pi3k is determined by a second pool of lck (lckp2) (see legend) which can be activated by tcr activation[61].
65	malt1 · card11 · bcl10 → card11a	1	The binding of malt1 to card11 and bcl10 forms the active card11 complex[7, 62, 63, 64].
66	mek → erk	1	mek phosphorylates erk leading to erk activation[11, 22].
67	mekk1 → jnk	1	mekk1 activates jnk[65].
68	mekk1 → mkk4	1	mekk1 is able to phosphorylate MKK4 leading to its activation[66].
69	mekk1 → p38	1	mekk1 leads to p38 activation[67].
70	mkk4 → jnk	1	MKK 4 activates jnk[65, 68].
71	mlk3 → mkk4	1	mlk3 phosphorylates mkk4[47].
72	nfkb →	1	nfkb is an output of the model.
73	p21c →	1	p21cip is an output of the model controlling the cell cycle.
74	p27k →	1	p27kip is an output of the model controlling the cell cycle.
75	p38 →	1	p38 is an output of the model.
76	p70s →	1	p70s is an output of the model.
77	pag → csk	1	Phosphorylation of pag allows csk to bind it and then act on lck[49, 17].
78	pdk1 → p70s	1	pkd1 phosphorylates p70s leading to its activation[69, 70].
79	pdk1 → pkb	1	pdk1 phosphorylates pkb leading to its activation[71, 72, 73].
80	pi3k · !ship1 · !pten → pip3	1	pi3k leads to the production of pip3, while ship1 and pten inhibit this process[74, 75].
81	pip3 → pdk1	1	pip3 is required for pdk1 activation[76].

Nr	Reaction	τ	Documentation
82	pip3 · zap70 · slp76 → itk	1	When phosphorylated, slp76 can bind to itk; additional binding to pip3 and phosphorylation via zap70 activates itk[11, 18, 77].
83	plcga → ip3	1	Active plcga splits pip2 into ip3 and diacylglycerol (dag,see hyperarc 11)[11, 18].
84	plcgb · !ccblp2 · slp76 · zap70 · vav1 · itk → plcga	1	Once bound to phosphorylated lat, plcgb is activated by the combined action of vav and itk (or rlk, see hyperarc 85)[77]. Additionally, binding to slp76 (phosphorylated by zap70) is required to establish and stabilize the complex. Activated ccbl degrades plcga[30].
85	plcgb · !ccblp2 · zap70 · vav1 · slp76 · rlk → plcga	1	Once bound to phosphorylated lat, plcgb is activated by the combined action of vav and rlk (or itk, see hyperarc 84)[77]. Additionally, binding to slp76 (phosphorylated by zap70) is required to establish and stabilize the complex. Activated ccbl degrades plcga[30].
86	rac1p1 → mlk3	1	Rac1p1 activates mlk3[78].
87	rac1p2 → mekk1	1	GTP-bound Rac1p2 is able to bind mekk1[36], and active mekk1 leads to JNK activation[79, 37].
88	rac1p2 → sre	1	Vav3-dependent Rac1 is able to activate Sre via SRF[80].
89	rac1r · vav1 → rac1p1	1	Downregulation of Vav1 but not Vav3 affects IL-2 production in T cells[81] via the rac1-mediated jnk pathway. Since rac1 mediates this process, we defined a vav1-dependent pool of rac1 (see hyperarc 90 and legend).
90	rac1r · vav3 → rac1p2	1	Downregulation of Vav3 but not Vav1 affects Sre activity[81]. Since rac1 mediates this process, we defined a vav3-dependent pool of rac1 (see hyperarc 89 and legend)
91	raf → mek	1	Raf phosphorylates mek leading to mek activation[82].
92	ras → raf	1	Ras mediates raf localization to the membrane, and consequently, raf is activated[22].
93	rsk → creb	1	Rsk phosphorylates creb increasing its activity[43].
94	sh3bp2 → vav3	1	sh3bp2 binds vav3 via an sh2 domain, leading to its activation[81].

Nr	Reaction	τ	Documentation
95	sos · !gap · rasgrp → ras	1	Bound to lat via grb2, sos catalyzes the exchange of GTP for GDP in the cellular-membrane-located ras, while rasgrp1 catalyzes the exchange of GTP for GDP in golgi-located ras[38]. In turn gap catalyzes the conversion GTP to GDP and thus deactivates ras[83].
96	sre →	1	Sre is an output of the model.
97	tcrb → dgk	2	dgks get activated after tcr activation in yet an unclear manner, we therefore make it dependent on activation of the tcr. Since dag must be produced in the early events, we assign it a τ=2[84].
98	tcrb · fyn → tcrp	1	Upon ligand binding to the tcr, active fyn can phosphorylate the tcr[85].
99	tcrb · lckp1 → tcrp	1	The co-localization of tcr with cd4 mediated by peptide-MHC or antibody crosslinking results in an increased local concentration of lck around the tcr leading to phosphorylation of ITAMs[52].
100	tcrb · lckr → fyn	1	A fraction of fyn is bound to the tcr, and tcr crosslinking leads to fyn auotophosphorylation and activation[85]. Since lck is required in the development for having capable fyn[86], lckr (existence of lck in the cell) is required as well.
101	zap70 → lat	1	zap70 phosphorylates lat at different sites[11].
102	zap70 · lat → sh3bp2	1	sh3bp2 binds to phosphorylated lat upon phosphorylation by zap70[87].
103	zap70 · sh3bp2 → vav1	1	zap70 phosphorylates vav1[81] which together with binding of vav1 to sh3bp2[87], leads to vav1 activation.
104	→ card11	1	Regulation of card11 is not clear, thus we set an external input to it. Default value is 1.
105	→ gadd45	1	Regulation of gadd45 is not clear, thus we set an external input to it. Default value is 1.
106	→ gap	1	GTP activating proteins (gaps) are important regulators of ras activation but their own regulation is not clear[88]. Therefore they are included in the model with an external input.
107	→ lckr	1	Input to the system (presence of Lck in the cell). Default value is 1.

Nr	Reaction	τ	Documentation
108	cam → camk2	1	cam (calmodulin) activates calmodulin-dependent kinase II (camk2) [89].
109	grb2 → sos	1	sos binds to grb2 and thus get recruited to the membrane via lat[90].
110	lat → gads	1	gads can bind to phosphorylated lat[18, 17].
111	cdc42 → sre	1	cdc42 is able to activate Sre via SRF[80].
112	nfat →	1	nfat is an output of the model.
113	shp2 →	1	shp2 is an output of the model.
114	→ cd45	1	Regulation of cd45 is not clear, thus we set an external input to it. Default value is 1.
115	→ pten	1	Regulation of pten is not clear, thus we set an external input. Default value is 0.
116	→ bcl10	1	Regulation of bcl10 is not clear, thus we set an external input to it. Default value is 1.
117	→ ccblr	1	Input to the system (presence of ccbl in the cell). Default value is 1.
118	→ cdc42	1	Regulation of cdc42 is not clear, thus we set an external input to it. Default value is 0.
119	→ malt1	1	Regulation of malt1 is not clear, thus we set an external input to it. Default value is 1.
120	→ rac1r	1	Input to the system (presence of rac1 in the cell). Default value is 1.
121	→ ship1	1	Regulation of ship1 is not clear, thus we set an external input. Default value is 0.
122	→ akap79	1	Regulation of akap79 is not clear, thus we set an external input to it. Default value is 0.
123	→ calpr1	1	Regulation of calpr1 is not clear, thus we set an external input to it. Default value is 0.

References

[1] Zha J, Harada H, Osipov K, Jockel J, Waksman G, et al. (1997) BH3 domain of BAD is required for heterodimerization with BCL-XL and pro-apoptotic activity. J Biol Chem 272:24101–24104.

[2] Yang E, Zha J, Jockel J, Boise LH, Thompson CB, et al. (1995) Bad, a heterodimeric partner for Bcl-XL and Bcl-2, displaces Bax and promotes cell death. Cell 80:285–291.

[3] Matsuda S, Shibasaki F, Takehana K, Mori H, Nishida E, et al. (2000) Two distinct action mechanisms of immunophilin-ligand complexes for the blockade of T-cell activation. EMBO Rep 1:428–434.

[4] Ryeom S, Greenwald RJ, Sharpe AH, McKeon F (2003) The threshold pattern of calcineurin-dependent gene expression is altered by loss of the endogenous inhibitor calcipressin. Nat Immunol 4:874–881.

[5] Kashishian A, Howard M, Loh C, Gallatin WM, Hoekstra MF, et al. (1998) AKAP79 inhibits calcineurin through a site distinct from the immunophilin-binding region. J Biol Chem 273:27412–27419.

[6] Pan F, Means AR, Liu JO (2005) Calmodulin-dependent protein kinase IV regulates nuclear export of Cabin1 during T-cell activation. EMBO J 24:2104–2113.

[7] Thome M (2004) Carma1, bcl-10 and malt1 in lymphocyte development and activation. Nat Rev Immunol 4:348–359. doi:10.1038/nri1352. URL http://dx.doi.org/10.1038/nri1352.

[8] Weil R, Israel A (2006) Deciphering the pathway from the TCR to NF-kappaB. Cell Death Differ 13:826–833.

[9] Hayden MS, Ghosh S (2004) Signaling to NF-kappaB. Genes Dev 18:2195–2224. doi:10.1101/gad.1228704. URL http://www.genesdev.org/cgi/content/abstract/18/18/2195. http://www.genesdev.org/cgi/reprint/18/18/2195.pdf.

[10] Khoshnan A, Bae D, Tindell CA, Nel AE (2000) The physical association of protein kinase C theta with a lipid raft-associated inhibitor of kappa B factor kinase (IKK) complex plays a role in the activation of the NF-kappa B cascade by TCR and CD28. J Immunol 165:6933–6940.

[11] Huang Y, Wange RL (2004) T cell receptor signaling: beyond complex complexes. J Biol Chem 279:28827–28830.

[12] Zipfel PA, Zhang W, Quiroz M, Pendergast AM (2004) Requirement for abl kinases in t cell receptor signaling. Current Biology 14:1222–1231. doi:10.1016/j.cub.2004.07.021. URL http://www.sciencedirect.com/science/article/B6VRT-4CY7JRM-N/2/6870c9cf19c500b35247fefa9d2f3347.

[13] Zhang J, Brdos T, Li D, Gl I, Vermes C, et al. (2002) Cutting edge: regulation of t cell activation threshold by cd28 costimulation through targeting cbl-b for ubiquitination. J Immunol 169:2236–2240. URL http://www.jimmunol.org/cgi/content/abstract/169/5/2236.

[14] Topham MK (2006) Signaling roles of diacylglycerol kinases. J Cell Biochem 97:474–484.

[15] Stefanova I, Dorfman JR, Germain RN (2002) Self-recognition promotes the foreign antigen sensitivity of naive t lymphocytes. Nature 420:429–434. doi:10.1038/nature01146. URL http://dx.doi.org/10.1038/nature01146.

[16] Altan-Bonnet G, Germain RNN (2005) Modeling t cell antigen discrimination based on feedback control of digital erk responses. PLoS Biol 3. doi:10.1371/journal.pbio.0030356. URL http://dx.doi.org/10.1371/journal.pbio.0030356.

[17] Horejsi V, Zhang W, Schraven B (2004) Transmembrane adaptor proteins: organizers of immunoreceptor signalling. Nat Rev Immunol 4:603–616. doi:10.1038/nri1414. URL http://dx.doi.org/10.1038/nri1414.

[18] Togni M, Lindquist J, Gerber A, Klsch U, Hamm-Baarke A, et al. (2004) The role of adaptor proteins in lymphocyte activation. Mol Immunol 41:615–630. doi:10.1016/j.molimm.2004.04.009. URL http://www.sciencedirect.com/science/article/B6T9R-4CFV5FC-6/2/af184e2559c33e3ebe106f7fb196fd40.

[19] Yamasaki S, Nishida K, Hibi M, Sakuma M, Shiina R, et al. (2001) Docking protein Gab2 is phosphorylated by ZAP-70 and negatively regulates T cell receptor signaling by recruitment of inhibitory molecules. J Biol Chem 276:45175–45183.

[20] Yamasaki S, Nishida K, Sakuma M, Berry D, McGlade CJ, et al. (2003) Gads/Grb2-mediated association with LAT is critical for the inhibitory function of Gab2 in T cells. Mol Cell Biol 23:2515–2529.

[21] Liang J, Slingerland JM (2003) Multiple roles of the pi3kpkb akt pathway in cell cycle progression by jiyong liang and joyce m. slingerland: Cell cycle article

abstract. Cell Cycle 2:339–345. URL http://www.landesbioscience.com/journals/cc/abstract.php?id=433.

[22] Krauss G (2003) Biochemistry of Signal Transduction and Regulation. Wiley-VCH.

[23] Hanada M, Feng J, Hemmings BA (2004) Structure, regulation and function of PKB/AKT–a major therapeutic target. Biochim Biophys Acta 1697:3–16.

[24] Salvador JM, Mittelstadt PR, Belova GI, Fornace AJ, Ashwell JD (2005) The autoimmune suppressor gadd45 inhibits the t cell alternative p38 activation pathway. Nature Immunology 6:396–402. doi:10.1038/ni1176. URL http://www.ncbi.nlm.nih.gov/entrez/query.fcgi?cmd=Retrieve\&db=pubmed\&dopt=Abstract\&list_uids=15735649.

[25] Palacios EH, Weiss A (2004) Function of the src-family kinases, lck and fyn, in t-cell development and activation. Oncogene 23:7990–8000. doi:10.1038/sj.onc.1208074. URL http://dx.doi.org/10.1038/sj.onc.1208074.

[26] Davidson D, Bakinowski M, Thomas ML, Horejsi V, Veillette A (2003) Phosphorylation-dependent regulation of t-cell activation by pag/cbp, a lipid raft-associated transmembrane adaptor. Mol Cell Biol 23:2017–2028. doi:10.1128/MCB.23.6.2017. URL http://dx.doi.org/10.1128/MCB.23.6.2017.

[27] Feske S, Giltnane J, Dolmetsch R, Staudt LM, Rao A (2001) Gene regulation mediated by calcium signals in T lymphocytes. Nat Immunol 2:316–324.

[28] Macian F (2005) NFAT proteins: key regulators of T-cell development and function. Nat Rev Immunol 5:472–484.

[29] Anderson K, Kane C (1998) Ca/calmodulin-dependent protein kinase iv and calcium signaling. Biometals 11:331 – 343.

[30] Rellahan BL, Graham LJ, Tysgankov AY, Debell KE, Veri MC, et al. (2003) A dynamic constitutive and inducible binding of c-cbl by plc[gamma]1 sh3 and sh2 domains (negatively) regulates antigen receptor-induced plc[gamma]1 activation in lymphocytes. Experimental Cell Research 289:184–194. doi:10.1016/S0014-4827(03)00260-X. URL http://www.sciencedirect.com/science/article/B6WFC-48TMBVK-3/2/126477995371634bb44f397e0908df2a.

[31] Rao N, Lupher ML, Ota S, Reedquist KA, Druker BJ, et al. (2000) The linker phosphorylation site tyr292 mediates the negative regulatory effect of cbl on zap-70 in t cells. J Immunol 164:4616–4626. URL http://www.ncbi.nlm.nih.gov/entrez/query.fcgi?cmd=Retrieve\&db=pubmed\&dopt=Abstract\&list_uids=10779765.

[32] Michel F, Mangino G, Attal-Bonnefoy G, Tuosto L, Alcover A, et al. (2000) CD28 utilizes Vav-1 to enhance TCR-proximal signaling and NF-AT activation. J Immunol 165:3820–3829.

[33] Raab M, Pfister S, Rudd CE (2001) Cd28 signaling via vav/slp-76 adaptors: regulation of cytokine transcription independent of tcr ligation. Immunity 15:921–933. URL http://www.ncbi.nlm.nih.gov/entrez/query.fcgi?cmd=Retrieve\&db=pubmed\&dopt=Abstract\&list_uids=11754814.

[34] Hehner SP, Hofmann TG, Dienz O, Droge W, Schmitz ML (2000) Tyrosine-phosphorylated vav1 as a point of integration for t-cell receptor- and cd28-mediated activation of jnk, p38, and interleukin-2 transcription. J Biol Chem 275:18160–18171. URL http://www.ncbi.nlm.nih.gov/entrez/query.fcgi?cmd=Retrieve\&db=pubmed\&dopt=Abstract\&list_uids=10849438.

[35] Kovacs B, Parry RV, Ma Z, Fan E, Shivers DK, et al. (2005) Ligation of cd28 by its natural ligand cd86 in the absence of tcr stimulation induces lipid raft polarization in human cd4 t cells. J Immunol 175:7848–7854.

[36] Fanger G, Johnson N, Johnson G (1997) Mek kinases are regulated by egf and selectively interact with rac/cdc42. EMBO J 16:4961–4972. URL http://www.pubmedcentral.nih.gov/articlerender.fcgi?artid=1170131.

[37] Kaga S, Ragg S, Rogers KA, Ochi A (1998) Activation of p21-cdc42/rac-activated kinases by cd28 signaling: p21-activated kinase (pak) and mek kinase 1 (mekk1) may mediate the interplay between cd3 and cd28 signals. J Immunol 160:4182–4189. URL http://www.ncbi.nlm.nih.gov/entrez/query.fcgi?cmd=Retrieve\&db=pubmed\&dopt=Abstract\&list_uids=9574518.

[38] Di Fiore PP (2003) Signal transduction: life on mars, cellularly speaking. Nature 424:624–625. doi:10.1038/424624a. URL http://dx.doi.org/10.1038/424624a.

[39] Bivona TG, De Castro PI, Ahearn IM, Grana TM, Chiu VK, et al. (2003) Phospholipase cgamma activates ras on the golgi apparatus by means of rasgrp1. Nature 424:694–698. doi:10.1038/nature01806. URL http://dx.doi.org/10.1038/nature01806.

[40] Roose JP, Mollenauer M, Gupta VA, Stone J, Weiss A (2005) A diacylglycerol-protein kinase c-rasgrp1 pathway directs ras activation upon antigen receptor stimulation of t cells. Mol Cell Biol 25:4426–4441. doi:10.1128/MCB.25.11.4426. URL http://www.ncbi.nlm.nih.gov/entrez/query.fcgi?cmd=Retrieve\&db=pubmed\&dopt=Abstract\&list_uids=15899849.

[41] Lee KY, D'Acquisto F, Hayden MS, Shim JH, Ghosh S (2005) Pdk1 nucleates t cell receptor-induced signaling complex for nf-kappab activation. Science 308:114–118. doi:10.1126/science.1107107. URL http://dx.doi.org/10.1126/science.1107107.

[42] Villalba M, Bi K, Hu J, Altman Y, Bushway P, et al. (2002) Translocation of pkc[theta] in t cells is mediated by a nonconventional, pi3-k- and vav-dependent pathway, but does not absolutely require phospholipase c. J Cell Biol 157:253–263. doi:10.1083/jcb.200201097. URL http://dx.doi.org/10.1083/jcb.200201097.

[43] Frdin M, Gammeltoft S (1999) Role and regulation of 90 kda ribosomal s6 kinase (rsk) in signal transduction. Mol Cell Endocrinol 151:65–77. URL http://www.ncbi.nlm.nih.gov/entrez/query.fcgi?cmd=Retrieve\&db=pubmed\&dopt=Abstract\&list_uids=10411321.

[44] Torgersen KM, Vang T, Abrahamsen H, Yaqub S, Horejs V, et al. (2001) Release from tonic inhibition of t cell activation through transient displacement of c-terminal src kinase (csk) from lipid rafts. J Biol Chem 276:29313–29318. doi:10.1074/jbc.C100014200. URL http://dx.doi.org/10.1074/jbc.C100014200.

[45] Arnaud M, Mzali R, Gesbert F, Crouin C, Guenzi C, et al. (2004) Interaction of the tyrosine phosphatase SHP-2 with Gab2 regulates Rho-dependent activation of the c-fos serum response element by interleukin-2. Biochem J 382:545–556.

[46] Hu MC, Qiu WR, Wang X, Meyer CF, Tan TH (1996) Human hpk1, a novel human hematopoietic progenitor kinase that activates the jnk/sapk kinase cascade. Genes Dev 10:2251–2264. URL http://www.ncbi.nlm.nih.gov/entrez/query.fcgi?cmd=Retrieve\&db=pubmed\&dopt=Abstract\&list_uids=8824585.

[47] Tibbles LA, Ing YL, Kiefer F, Chan J, Iscove N, et al. (1996) MLK-3 activates the SAPK/JNK and p38/RK pathways via SEK1 and MKK3/6. EMBO J 15:7026–7035.

[48] Chan TO, Rittenhouse SE, Tsichlis PN (1999) AKT/PKB and other D3 phosphoinositide-regulated kinases: Kinase activation by phosphoinositide-dependent phosphorylation. Annu Rev Biochem 68:965–961014.

[49] Lindquist JA, Simeoni L, Schraven B (2003) Transmembrane adapters: attractants for cytoplasmic effectors. Immunol Rev 191:165–182. URL http://www.ncbi.nlm.nih.gov/entrez/query.fcgi?cmd=Retrieve\&db=pubmed\&dopt=Abstract\&list_uids=12614359.

[50] Liou J, Kiefer F, Dang A, Hashimoto A, Cobb MH, et al. (2000) Hpk1 is activated by lymphocyte antigen receptors and negatively regulates ap-1. Immunity 12:399–408. doi:10.1016/S1074-7613(00)80192-2. URL http://www.sciencedirect.com/science/article/B6WSP-41BD5XG-5/2/4221b7315158bbba66b8e3184f0f3fe8.

[51] Shan X, Czar MJ, Bunnell SC, Liu P, Liu Y, et al. (2000) Deficiency of pten in jurkat t cells causes constitutive localization of itk to the plasma membrane and hyperresponsiveness to cd3 stimulation. Mol Cell Biol 20:6945–6957. URL http://www.ncbi.nlm.nih.gov/entrez/query.fcgi?cmd=Retrieve\&db=pubmed\&dopt=Abstract\&list_uids=10958690.

[52] Filipp D, Leung BL, Zhang J, Veillette A, Julius M (2004) Enrichment of lck in lipid rafts regulates colocalized fyn activation and the initiation of proximal signals through tcr alpha beta. J Immunol 172:4266–4274. URL http://www.ncbi.nlm.nih.gov/entrez/query.fcgi?cmd=Retrieve\&db=pubmed\&dopt=Abstract\&list_uids=15034040.

[53] Deane JA, Fruman DA (2004) Phosphoinositide 3-kinase: Diverse roles in immune cell activation. Annu Rev Immunol 22:563–598.

[54] Fang D, Liu YC (2001) Proteolysis-independent regulation of PI3K by Cbl-b-mediated ubiquitination in T cells. Nat Immunol 2:870–875.

[55] August A, Dupont B (1994) CD28 of T lymphocytes associates with phosphatidylinositol 3-kinase. Int Immunol 6:769–774.

[56] Ghiotto-Ragueneau M, Battifora M, Truneh A, Waterfield MD, Olive D (1996) Comparison of CD28-B7.1 and B7.2 functional interaction in resting human T cells: Phosphatidylinositol 3-kinase association to CD28 and cytokine production. Eur J Immunol 26:34–41.

[57] von Willebrand M, Baier G, Couture C, Burn P, Mustelin T (1994) Activation of phosphatidylinositol-3-kinase in Jurkat T cells depends on the presence of the p56lck tyrosine kinase. Eur J Immunol 24:234–238.

[58] Gibson S, Truitt K, Lu Y, Lapushin R, Khan H, et al. (1998) Efficient CD28 signalling leads to increases in the kinase activities of the TEC family tyrosine kinase EMT/ITK/TSK and the SRC family tyrosine kinase LCK. Biochem J 330 (Pt 3):1123–1128.

[59] Holdorf AD, Green JM, Levin SD, Denny MF, Straus DB, et al. (1999) Proline residues in CD28 and the Src homology (SH)3 domain of Lck are required for T cell costimulation. J Exp Med 190:375–384.

[60] Huang J, Lo PF, Zal T, Gascoigne NRJ, Smith BA, et al. (2002) CD28 plays a critical role in the segregation of PKC theta within the immunologic synapse. Proc Natl Acad Sci U S A 99:9369–9373.

[61] Okkenhaug K, Vanhaesebroeck B (2003) PI3K in lymphocyte development, differentiation and activation. Nat Rev Immunol 3:317–330.

[62] Gaide O, Favier B, Legler DF, Bonnet D, Brissoni B, et al. (2002) Carma1 is a critical lipid raft-associated regulator of tcr-induced nf-kappa b activation. Nat Immunol 3:836–843. doi:10.1038/ni830. URL http://dx.doi.org/10.1038/ni830.

[63] Uren AG, O'Rourke K, Aravind L, Pisabarro TM, Seshagiri S, et al. (2000) Identification of paracaspases and metacaspases: Two ancient families of caspase-like proteins, one of which plays a key role in malt lymphoma. Molecular Cell 6:961–967. doi:10.1016/S1097-2765(05)00086-9. URL http://www.sciencedirect.com/science/article/B6WSR-4CYGMTG-3/2/12fca308fb91a72a6201f47c1a98b063.

[64] Lucas PC, Yonezumi M, Inohara N, McAllister-Lucas LM, Abazeed ME, et al. (2001) Bcl10 and malt1, independent targets of chromosomal translocation in malt lymphoma, cooperate in a novel nf-kappa b signaling pathway. J Biol Chem 276:19012–19019. doi:10.1074/jbc.M009984200. URL http://www.jbc.org/cgi/content/abstract/276/22/19012.

[65] Davis RJ (2000) Signal transduction by the jnk group of map kinases. Cell 103:239–252. URL http://www.ncbi.nlm.nih.gov/entrez/query.fcgi?cmd=Retrieve\&db=pubmed\&dopt=Abstract\&list_uids=11057897.

[66] Yan M, Dai T, Deak JC, Kyriakis JM, Zon LI, et al. (1994) Activation of stress-activated protein kinase by MEKK1 phosphorylation of its activator SEK1. Nature 372:798–800.

[67] Guan Z, Buckman SY, Pentland AP, Templeton DJ, Morrison AR (1998) Induction of cyclooxygenase-2 by the activated mekk1 right-arrowsek1/mkk4 right-arrowp38 mitogen-activated protein kinase pathway. J Biol Chem 273:12901–12908. URL http://www.ncbi.nlm.nih.gov/entrez/query.fcgi?cmd=Retrieve\&db=pubmed\&dopt=Abstract\&list_uids=9582321.

[68] Dérijard B, Raingeaud J, Barrett T, Wu IH, Han J, et al. (1995) Independent human MAP-kinase signal transduction pathways defined by MEK and MKK isoforms. Science 267:682–685.

[69] Pullen N, Dennis PB, Andjelkovic M, Dufner A, Kozma SC, et al. (1998) Phosphorylation and activation of p70s6k by PDK1. Science 279:707–710.

[70] Hinton HJ, Alessi DR, Cantrell DA (2004) The serine kinase phosphoinositide-dependent kinase 1 (PDK1) regulates T cell development. Nat Immunol 5:539–545.

[71] Lafont V, Astoul E, Laurence A, Liautard J, Cantrell D (2000) The t cell antigen receptor activates phosphatidylinositol 3-kinase-regulated serine kinases protein kinase b and ribosomal s6 kinase 1. FEBS Letters 486:38–42. doi:10.1016/S0014-5793(00)02235-3. URL http://www.sciencedirect.com/science/article/B6T36-41SSXC9-9/2/50f6f5a78abd403ca5f876d11bcabb7a.

[72] Alessi DR, James SR, Downes CP, Holmes AB, Gaffney PR, et al. (1997) Characterization of a 3-phosphoinositide-dependent protein kinase which phosphorylates and activates protein kinase Balpha. Curr Biol 7:261–269.

[73] Anderson KE, Coadwell J, Stephens LR, Hawkins PT (1998) Translocation of PDK-1 to the plasma membrane is important in allowing PDK-1 to activate protein kinase B. Curr Biol 8:684–691.

[74] Okkenhaug K, Bilancio A, Emery JL, Vanhaesebroeck B (2004) Phosphoinositide 3-kinase in T cell activation and survival. Biochem Soc Trans 32:332–335.

[75] Rameh LE, Cantley LC (1999) The role of phosphoinositide 3-kinase lipid products in cell function. J Biol Chem 274:8347–8350. URL http://www.ncbi.nlm.nih.gov/entrez/query.fcgi?cmd=Retrieve\&db=pubmed\&dopt=Abstract\&list_uids=10085060.

[76] Mora A, Komander D, van Aalten DMF, Alessi DR (2004) PDK1, the master regulator of AGC kinase signal transduction. Semin Cell Dev Biol 15:161–170.

[77] Czar MJ, Debnath J, Schaeffer EM, Lewis CM, Schwartzberg PL (2001) Biochemical and genetic analyses of the tec kinases itk and rlk/txk. Biochem Soc Trans 29:863–867. URL http://www.ncbi.nlm.nih.gov/entrez/query.fcgi?cmd=Retrieve\&db=pubmed\&dopt=Abstract\&list_uids=11709089.

[78] Teramoto H, Coso OA, Miyata H, Igishi T, Miki T, et al. (1996) Signaling from the small GTP-binding proteins Rac1 and Cdc42 to the c-Jun N-terminal kinase/stress-activated protein kinase pathway. A role for mixed lineage kinase 3/protein-tyrosine kinase 1, a novel member of the mixed lineage kinase family. J Biol Chem 271:27225–27228.

[79] Minden A, Lin A, Claret FX, Abo A, Karin M (1995) Selective activation of the JNK signaling cascade and c-Jun transcriptional activity by the small GTPases Rac and Cdc42Hs. Cell 81:1147–1157.

[80] Hill CS, Wynne J, Treisman R (1995) The Rho family GTPases RhoA, Rac1, and CDC42Hs regulate transcriptional activation by SRF. Cell 81:1159–1170.

[81] Zakaria S, Gomez TS, Savoy DN, McAdam S, Turner M, et al. (2004) Differential regulation of TCR-mediated gene transcription by Vav family members. J Exp Med 199:429–434.

[82] Franklin RA, Tordai A, Patel H, Gardner AM, Johnson GL, et al. (1994) Ligation of the t cell receptor complex results in activation of the ras/raf-1/mek/mapk cascade in human t lymphocytes. J Clin Invest 93:2134–2140. URL `http://www.ncbi.nlm.nih.gov/entrez/query.fcgi?cmd=Retrieve\&db=pubmed\&dopt=Abstract\&list_uids=8182145`.

[83] Genot E, Cantrell DA (2000) Ras regulation and function in lymphocytes. Curr Opin Immunol 12:289–294.

[84] Sanjuan MA, Jones DR, Izquierdo M, Merida I (2001) Role of Diacylglycerol Kinase alpha in the Attenuation of Receptor Signaling. J Cell Biol 153:207–220. doi:10.1083/jcb.153.1.207. URL `http://www.jcb.org/cgi/content/abstract/153/1/207`.

[85] Filipp D, Julius M (2004) Lipid rafts: resolution of the ”fyn problem”? Mol Immunol 41:645–656. doi:10.1016/j.molimm.2004.04.011. URL `http://dx.doi.org/10.1016/j.molimm.2004.04.011`.

[86] Zamoyska R, Basson A, Filby A, Legname G, Lovatt M, et al. (2003) The influence of the src-family kinases, Lck and Fyn, on T cell differentiation, survival and activation. Immunol Rev 191:107–118.

[87] Qu X, Kawauchi-Kamata K, Miah SMS, Hatani T, Yamamura H, et al. (2005) Tyrosine phosphorylation of adaptor protein 3BP2 induces T cell receptor-mediated activation of transcription factor. Biochemistry 44:3891–3898.

[88] Cantrell DA (2003) Gtpases and t cell activation. Immunol Rev 192:122–130. URL `http://www.ncbi.nlm.nih.gov/entrez/query.fcgi?cmd=Retrieve\&db=pubmed\&dopt=Abstract\&list_uids=12670400`.

[89] Hughes K, Edin S, Antonsson A, Grundstrm T (2001) Calmodulin-dependent kinase ii mediates t cell receptor/cd3- and phorbol ester-induced activation of ikappab kinase. J Biol Chem 276:36008–36013. doi:10.1074/jbc.M106125200. URL `http://www.jbc.org/cgi/content/abstract/276/38/36008`.

[90] Buday L, Egan SE, Rodriguez Viciana P, Cantrell DA, Downward J (1994) A complex of grb2 adaptor protein, sos exchange factor, and a 36-kda membrane-bound tyrosine phosphoprotein is implicated in ras activation in t cells. J Biol Chem 269:9019–9023. URL http://www.ncbi.nlm.nih.gov/entrez/query.fcgi?cmd=Retrieve\&db=pubmed\&dopt=Abstract\&list_uids=7510700.
